# Commentary: The impact of iron deposition on the fear circuit of the brain in patients with Parkinson's disease and anxiety

**DOI:** 10.3389/fnagi.2023.1223421

**Published:** 2023-06-19

**Authors:** Leilei Chen, Junxia Xie

**Affiliations:** Institute of Brain Science and Disease, Shandong Provincial Collaborative Innovation Center for Neurodegenerative Disorders, Shandong Provincial Key Laboratory of Pathogenesis and Prevention of Neurological Disorders, Qingdao University, Qingdao, China

**Keywords:** Parkinson's disease, anxiety, iron deposition, fear circuit, QSM

Parkinson's disease (PD) is a common neurodegenerative disorder which affects more that 1% of the population over the age of 60. The major pathological features of PD include selective dopaminergic neuron loss in the substantia nigra, abnormal α-synuclein aggregation, iron deposition and so on. Under normal physiological conditions, iron will be deposited in brain nuclei, such as substantia nigra, caudate nucleus and globus pallidus with increasing age, however, iron deposition is more serious in PD (Mcallum et al., 2020). In recent years, with the in-depth investigations on PD, abnormal iron accumulation is considered to be one of the most important mechanisms of nigral neurodegeneration in PD. Quantitative susceptibility mapping (QSM) is a sensitive magnetic resonance imaging (MRI) technique, which has been recently widely used to quantify susceptibility changes in brain. QSM data has reported significant iron deposition in bilateral substantia nigra in patients with idiopathic rapid eye movement sleep behavior disorder, which is an early sign of neurodegenerative disease and about 33.8% of clinically diagnosed patients with idiopathic rapid eye movement sleep behavior disorder developed neurodegenerative disease within 4 years (Sun et al., 2019), suggesting that abnormal nigral iron deposition may accelerate neurodegenerative diseases from prodromal stage to clinical stage. Also, the nigral iron level has been reported to be correlated positively with the disease duration, motor and cognitive dysfunction in PD patients (Tambasco et al., 2019). Most of the existing researches have focused on the nigral iron deposition, however, investigations about the level and role of iron in other brain regions are still limited in PD. As a response to prospective or imagined future danger or threat, anxiety is a common but often under-recognized neuropsychiatric manifestations of PD, which is considered to share the fear circuit, including hippocampus, medial prefrontal cortex (mPFC), amygdala (AMG), anterior cingulate cortex (ACC), insula, and striatum, with fear (Penninx et al., 2021). Structural modifications of the left AMG, over-activation of the AMG, as well as impaired mPFC and insula have been identified by functional MRI in PD patients with anxiety (Carey et al., 2020; Criaud et al., 2021), and dysfunction of the fear circuit is suggested to be associated with the anxiety in PD. Although the important role of iron deposition has been demonstrated in the development of PD, there is no report about the change of iron levels in the fear circuit of PD.

Recently, the report by Chen et al. (2023), which is titled “The impact of iron deposition on the fear circuit of the brain in patients with Parkinson's disease and anxiety,” investigated the alteration in brain iron deposition in the fear circuit of PD patients with anxiety. Totally, thirty-nine PD patients and twenty-six age-and sex-matched healthy controls were enrolled in the assessment study, which has been divided into three groups: PD patients with anxiety (PD-A, *n* = 16), PD patients without anxiety (PD-NA, *n* = 23), and healthy controls (HC, *n* = 26). Hamilton Anxiety Rating Scale (HAMA) was employed to evaluate the mental state of PD patients. In the PD-A group, PD patients should meet two rules, one of which is that PD patients meet the diagnostic criteria of anxiety as defined by the DSM-IV (Diagnostic and Statistical Manual of Mental Disorders, fourth edition) criteria, the other one of which is that the HAMA scores ≥ 12. Voxel-based QSM analysis was employed to compare the susceptibility of the whole brain among the three groups. And then, regional QSM values related to fear circuit were obtained based on the region of interest (ROI)-based analysis, and the correlation between the regional QSM values in the fear circuit and anxiety scores was also analyzed. The voxel-based QSM analysis revealed that, compared to the HC group, increased QSM values, which indicate increased iron level, were observed in several brain regions of PD-A group [including ventral mPFC, ventral ACC, precuneus, angular gyrus, middle occipital gyrus, and supplementary motor area (SMA)] and PD-NA group (including parahippocampal gyrus and superior temporal gyrus). Notably, compared to the PD-NA and HC groups, significantly higher QSM values was observed in the hippocampus of PD-A group. Correlation analysis revealed that the QSM values of the hippocampus (*r* = 0.496, *p* < 0.01), mPFC (*r* = 0.255, *p* < 0.04), and ACC (*r* = 0.381, *p* < 0.01) are positively correlated with HAMA scores. In addition, compared to the HC group, significantly increased QSM values were observed in the substantia nigra (PD-A: *p* < 0.01; PD-NA: *p* < 0.01) and globus pallidus (PD-A: *p* = 0.024; PD-NA: *p* = 0.034) of PD patients. This is a first report that increased iron accumulation in the fear circuit in PD patients with anxiety, which highlight that abnormal iron deposition in the related brain regions might contribute to the development of anxiety in PD.

In addition to QSM, transcranial sonography (TCS) is also a non-invasive method for detecting iron deposition in the midbrain. It has been suggested that the parameters of TSC and MRI should be considered complementary in the evaluation of iron deposition in PD (Zhu et al., 2017; Prasuhn et al., 2022). Also, in the study of anxiety-related QSM ROI, the factors of depression and disease course were not excluded as covariates by Chen et al., which should be noted in future studies. Previously, two axonal iron transport pathways in the brain has been reported, one of which is from ventral hippocampus to mPFC to substantia nigra; and the other one of which is from thalamus (Tha) to AMG to mPFC (Wang et al., 2019), and abnormal axonal iron transport among brain regions might contribute to the nigral iron deposition in PD (Chen et al., 2021). Interestingly, the iron transport from ventral hippocampus to mPFC modulated anxiety-related behaviors, and the promotion of iron transport from ventral hippocampus to mPFC by genetic or pharmacological manner could produce anxiolytic-like effects (Wang et al., 2019). Considering the observations by Chen et al., abnormal axonal iron transport form ventral hippocampus to mPFC might be also involved in the anxiety of PD. In addition, changes of iron related proteins, which response for the iron import, iron storage and iron export, might occur in these brain regions of fear circuit during the development of anxiety in PD. Ferroptosis is an iron dependent cell death form, which has attacked more and more attentions in PD pathogenesis (Chen and Xie, 2020; Jiang et al., 2023). Further investigations focused on the ferroptosis in these iron deposited brain regions of fear circuit may yield new mechanism and perspective for the development of anxiety in PD. As disturbances of calcium homeostasis have also been indicated to be associated with the pathological development of PD (Zhang et al., 2022), it is also worthy investigating the imbalance of calcium in the fear circuit of PD patients or PD animal models. Taken together, this study about the abnormal iron accumulation in the fear circuit provides a new direction to explain the anxiety in PD, and further investigations addressing the above points might yield new mechanism for the PD pathology.

## Author contributions

LC wrote the first draft of the manuscript. Both authors contributed to manuscript revision, read, and approved the submitted version.
